# Exploring clinician perceptions of a care pathway for the management of shoulder pain: a qualitative study

**DOI:** 10.1186/s12913-022-07999-z

**Published:** 2022-05-25

**Authors:** Leslie Verville, Carol Cancelliere, Gaelan Connell, Joyce Lee, Silvano Mior, Sarah Munce, Robin Kay, Pierre Côté

**Affiliations:** 1 University of Ontario Institute of Technology (Ontario Tech University), Oshawa, Ontario Canada; 2 Canadian Memorial Chiropractic College, Toronto, Ontario Canada; 3 KITE-Toronto Rehabilitation Institute, University Health Network, Toronto, Ontario Canada

**Keywords:** Integrated knowledge translation, Clinical care pathway, Qualitative inquiry

## Abstract

**Background:**

Clinical care pathways may be useful tools to improve the quality of healthcare by facilitating the translation of evidence into practice. Our study is situated within a larger project, whereby end-users co-developed a care pathway for the management of shoulder pain. In this study, we explored end-user perceptions of the usefulness and practicality of implementing a care pathway to manage shoulder pain. We also solicited feedback for the pathway’s improvement.

**Methods:**

We conducted a qualitative study using a transcendental phenomenological approach seen through a constructivist lens. Clinicians recorded themselves interacting with the care pathway while working through a clinical case. Clinicians described their thoughts and movements aloud as they completed the activity. Second, we conducted individual semi-structured interviews to discuss the usefulness and practicality of pathway implementation. Interview transcripts were coded independently by reviewers. Transcript codes and associated quotes were grouped into themes. Themes were sequenced and linked creating a ‘web’ of thematic connections. Summary statements were developed to synthesize the overall essence of the phenomena.

**Results:**

Nine clinicians participated. Participants included eight chiropractors and one medical physician. We found that clinicians believed the care pathway could be useful at various levels, including education (students, interns), for early career clinicians, for engaging patients, facilitating interprofessional communication, and as a reminder of information for certain, less familiar conditions. When discussing the practicality of implementing the care pathway into practice settings, clinicians expressed that agreement with the care pathway and its recommendations may influence its acceptability among clinicians. Additionally, integrating recommendations into practice may be a skill requirement included into clinical training. Clinicians described the importance of opinion leaders in the acceptability of new evidence. Various difficulties with the replicability of interventions into clinical care was also discussed. In general, clinicians suggested the layout of the care pathway was manageable, and there was sufficient information for clinical decision-making. Clinicians also made several recommendations for improvement.

**Conclusions:**

End-user involvement and collaboration provides tangible instruction to improve care pathways themselves, their implementation strategies and helps to support and strengthen future research for overcoming individual, systemic and contextual barriers.

**Supplementary Information:**

The online version contains supplementary material available at 10.1186/s12913-022-07999-z.

## Background

The translation of research into practice is often slow, incomplete, and inconsistent [1]. The lag time between research development and its uptake into practice results in the possibility that patients may not be receiving the most beneficial, up-to-date care possible. Clinical care pathways may be useful tools to help fill this gap and facilitate the translation of research into practice.

Clinical care pathways are structured healthcare management tools designed to provide consistent, up-to-date evidence-based care [2]. In addition, care pathways support the mutual decision-making between patients and clinicians [2]. Conceptually, the growing body of literature supports the use of care pathways; however, their development and dissemination alone are not enough to meaningfully impact practice behaviour [3, 4]. Significant individual, systems, and contextual barriers along with awareness and lack of familiarity may hinder the uptake of care pathways and associated clinical recommendations in practice settings [5–7]. For instance, hesitancy to implement new evidence may be due to limited organizational support, conflicting information between sets of guidelines, and reluctance to change currently offered interventions to patients, especially when there appears to be anecdotal evidence of effectiveness [8]. Furthermore, clinicians may be hesitant to implement clinical care pathways because of perceived loss of autonomy over their clinical decision-making [4, 9, 10].

In an effort to appeal to end-users, clinical care pathways are developed in collaboration and/or consultation with end-users, with consideration of evidence-based recommendations and context-specific elements [5]. Engaging with end-users throughout the development of the care pathway may provide helpful insights about these specific barriers, as well as strategies to overcome them [6, 11–13]. This engagement and collaboration may be facilitated by employing integrated Knowledge Translation (iKT) strategies. iKT is a well-supported approach to conducting research that involves integrating knowledge users throughout the entire research process alongside the researchers, thereby maximizing the accessibility, relevance, and endurance of research outcomes [14–20].

Our study is situated within a larger project, whereby end-users (i.e., clinicians) co-developed an evidence-based practice tool with our team of researchers [21]. The practice tool is an online care pathway that aims to facilitate the clinical management of soft-tissue shoulder pain in adults. The practice tool was developed based upon the recommendations and clinical pathway described in the clinical guideline for the management of soft tissue shoulder injuries by Yu et al. [22]. While we used this guideline for the content of the care pathway tool, it was not the primary focus of our study. Our primary interest was the tool itself; we envision the content could be substituted with other guideline information in future iterations. For the purposes of this study, we engaged end-users to gain an understanding of their needs and preferences (e.g., current practice patterns, knowledge gaps, information trends, preferred tool formatting), as well as barriers to its implementation (e.g., patient, professional, organizational, system, economic, political, or social/cultural factors). In this current study, our objectives were to 1) explore end-user perceptions of the usefulness of the care pathway; 2) explore end-user perceptions about the practicality of implementing the care pathway into practice settings; and 3) describe end-user feedback and recommendations for improving the care pathway.

## Methods

### Study design

**Fig. 1:**
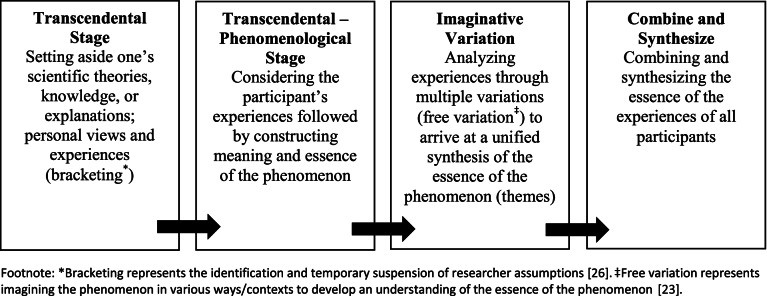
Transcendental phenomenological approach

We conducted a qualitative study using a transcendental phenomenological approach seen through a constructivist lens (Fig. 1) [23–26]. This approach aims to understand the essence of a phenomenon whereby the researcher seeks to achieve a bias-free state of mind and relies on the objective interpretation of participants’ lived experiences [23].

We obtained ethics approval through the Research Ethics Board of Ontario Tech University (REB #15436). We used the Consolidated Criteria for Reporting Qualitative Research (COREQ) to guide this study’s conduct and reporting. The methods of this study have been previously reported in a related study [21].

### Participants and recruitment

We invited clinicians, from our professional networks, with disciplines that commonly manage patients with shoulder pain (i.e., chiropractors, medical physicians, physiotherapists) to co-develop a care pathway with our team of researchers, and then participate in a semi-structured interview to address our current research objectives. We used purposeful maximum variation sampling to increase generalizability to achieve diversity in years of practice, discipline, practice characteristics (multidisciplinary vs. solo), and geographical representation (Canada, United States). Clinicians were contacted via email and sent an invitation letter. Recruitment occurred between May 2020 and October 2020 until data saturation was reached, defined as the point at which no new helpful information relative to our study objectives was obtained for two consecutive interviews [27].

### Care pathway

**Fig. 2:**
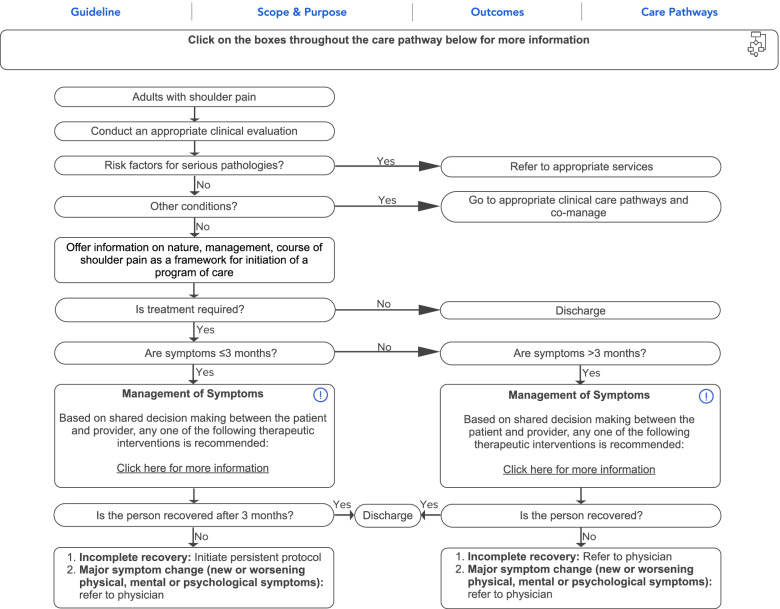
Care pathway for the management of shoulder pain

Before the interviews, we developed an online care pathway for managing shoulder pain based on the clinical practice guideline on the non-invasive management of soft tissue disorders of the shoulder (Fig. 2) [22]. The care pathway tool included information about the shoulder guideline [22], its scope and purpose, and links to outcome measurement tools. The care pathway also included essential aspects of the clinician-patient interaction, including components of the clinical evaluation and treatment recommendations. Each decision or statement in the care pathway flowchart was hyperlinked that when selected provided more detailed information or definition.

### Think aloud activity

Clinicians were asked to use a software called ‘Loom’ to create audio and video recordings of their interaction with the care pathway and the clinical case [28]. A clinical case study on the management of shoulder pain was randomly assigned to each clinician and sent to them prior to the recording. Each case provided details about a patient from the beginning of an interaction to the treatment and follow-up period. Clinicians were instructed to describe their thoughts and movements aloud as they complete the activity.

Once they completed the activity, they were asked to answer the following questions: (1) what did you like about the care pathway? (2) what didn’t you like about the care pathway? (3) was there enough information in the care pathway to inform your clinical decision-making? (4) how would you improve the care pathway? and (5) how would you use the care pathway in your practice? Their answers were also captured in the audio and video recordings using ‘Loom’. The recorded responses from the Think Aloud activity were analyzed to inform the recommendations to improve the care pathway.

### Semi-structured interviews

After developing the care pathway and completing the Think Aloud activity, we conducted individual semi-structured interviews using Zoom v.5.0. Interviews were used to explore participants’ perceptions about the usefulness of the care pathway (research objective 1) and their perceptions about the practicality of its implementation in clinical practice (research objective 2). We also collected feedback on participants’ likes, dislikes, and suggestions for improving the care pathway tool (research objective 3). We used a semi-structured interview methodology because of its versatility and flexibility, enabling reciprocity between the interviewer and interviewee, enabling a more natural flow of conversation while ensuring key points were explored [28, 29].

A trained interviewer led all interviews (GC) [credentials: BHK, DC; occupation: research associate; sex: male]. An additional female researcher was present during each interview (LV, JL). Their role was to support the primary interviewer to ensure conversation remained in-line with the interview guide, when necessary, and write field notes. The interviewers and participants knew each other within a professional capacity. Participants were unaware of the interview questions before the interview (Additional file 1). Interviews lasted approximately 45 minutes and were audio-recorded. Recordings were saved as audio files and transcribed verbatim by an external transcriptionist. Following each interview, clinicians were offered an opportunity to review and correct/clarify their transcripts before analysis (i.e., member checking). This was done to ensure clinicians felt that their responses to the interview questions were accurately portrayed [30]. We conducted only one round of interviews per participant. Personal identifying information was removed from the transcripts prior to analysis.

### Analysis

We used a transcendental phenomenological approach to conduct our identifying analysis from the interviews. In addition, we adopted the six-phase conceptual framework by Braun and Clark [30] to support our approach: 1) familiarizing yourself with the data; 2) generating initial codes; 3) searching for themes; 4) reviewing themes; 5) defining and naming themes, and 6) producing the report [31]. While this framework is presented in phases, this process was not conducted linearly. We revisited phases throughout the analysis process to facilitate a thorough interpretation of the data [31].

An initial open coding manual with definitions was developed a priori based on our research objectives and the interview guide to identifying major categories (themes) of information (Table 1) [32]. Throughout the analysis process, new codes were added, and existing codes were modified and refined to develop a complete and representative coding scheme.

**Table 1:** Organizational coding tree and definitions

Theme	Definition
Perceived usefulness to clinicians	Pertaining to the use or perceived use of the care pathway in practice
Practicality	Pertaining to barriers or enablers (real or perceived) of implementing the care pathway into practice
Feedback	Pertaining to any design or content feedback that could be used to improve the care pathway

Transcripts were analyzed throughout the data collection period. Ongoing transcript analysis allowed us to update our interview guide iteratively, ensure we met our research objectives’ needs, and determine when we had reached saturation of themes. Following each interview, three reviewers (LV, GC, JL) independently coded the transcript. Independent coding of transcripts was used to ensure reliability of interpretation. Field notes were used to provide contextualization of the transcript quotes when necessary. Reviewers then met as a group to compare coding and reach consensus through discussion. Throughout the analysis, the coding scheme and definitions were revised as reviewers attempted to construct meaning about the phenomenon [32]. Subsequent transcripts were coded using the continually revised coding scheme. Once the coding scheme was revised, previous transcripts were re-coded (LV) according to the updated scheme. This process was continued until all transcripts were coded. Saturation was assessed following the consensus of each transcript and was determined when no new themes emerged for two consecutive interviews.

We used NVIVO v.11 to organize the codes and quotes from the interview transcripts (conducted by LV; checked by GC, JL). We created mind maps [33, 34] with headings representing our three research objectives: clinician perception of usefulness (1) clinician perceptions of the practicality of implementation (2) and feedback to improve the care pathway (3). Transcript codes and associated quotes were grouped into themes. Broad themes were linked to sub-themes to describe overall phenomena related to our objectives. Themes were sequenced and linked to one another, creating a ‘web’ of thematic connections. We implemented member checking to improve the trustworthiness of our analysis by asking clinicians to review and provide feedback about the mind maps, themes, and definitions to ensure that our interpretation of the comments was appropriate [35]. Summary statements were developed to synthesize the overall essence of the phenomena for each research objectives supported by themes and clinician quotes. We selected representative quotes that most articulately described each theme.

## Results

Eighteen clinicians were invited to participate (9 chiropractors, 3 physiotherapists, and 6 medical physicians); of those, 12 consented, and nine participated. We were unable to obtain reasons for non-participation. Saturation of themes was achieved within the participant sample.

Of the clinicians who participated, eight were chiropractors (89%) and one was a medical physician. No physiotherapists participated. Most clinicians were male (89%). Age ranged from (27–77 years); with a mean age of 46 years (SD 15.2). Most clinicians practiced in Canada (8/9). Years in practice ranged from (2–45 years) with a mean of 16 years in practice (SD 13). Most professional degrees were attained in Canada (78%); 67% of clinicians reported attaining at least one additional advanced degree (e.g., Master’s degree, Ph.D.).

We invited all participants to review their transcripts and resultant findings. One participant edited their transcript, and five provided edits to the mind maps, themes, and definitions.

### Research objective 1: end-user perceptions of usefulness of the online care pathway

We aimed to understand whether clinicians thought the care pathway was useful in practice and who they perceived would use it. Three themes emerged from exploring clinicians’ perceptions of usefulness of the online care pathway.

### Theme: clinical applicability of the care pathway

Clinicians indicated that the care pathway could be a useful educational tool for students, interns, and novice practitioners. Specifically, the clinician participants perceived the care pathway as more appropriate and of greater benefit to novice clinicians than experienced clinicians. For example:

“Certainly, having access to tools to help me in my practice better assess and treat shoulders would be beneficial because it is not something that I see every day and so as someone who is a new grad I am still trying to gain experience and that experience might come over a good length of time. If I were to have tools and evidence-based pathways to help me, I think it would be more beneficial, and help me gain that experience and gain my confidence in treating those conditions.” –Clinician 8 (male; 2 years in practice)

Conversely, more experienced clinicians, with previous experience managing shoulder conditions, suggested they would not likely refer to the care pathway for assistance. Clinicians also expressed that because the care pathway did not highlight any new or changing evidence from what they already knew, they would be less likely to use the care pathway.

“…the reality of it is, our colleagues would already believe that they have this sorted out. So no need to take a look at this, I’ve got this one covered; which is why I think you were again right to point out that if there was some change or some new research or some new way or approach or new way of thinking about this that was different than the way that you would typically practice then you would for sure have an upswing in people wanting to engage this material. In the absence of that, people would be like yeah, yeah, I got no problems on shoulders.” – Clinician 3 (male; 20 years in practice)

In addition, it was expressed that the care pathway may be useful as a refresher or reminder for certain topics they may be less familiar or use it as a tool to confirm their understanding and knowledge of the current evidence.

“I am not so sure these are created for people to use with every single person but I think they are meant to just be a brush up sometimes for people to refer back to.” –Clinician 2 (male; 16 years in practice)

### Theme: facilitating communication

Some clinicians expressed that they envision the care pathway as a useful tool for improving or facilitating collaborative- and interprofessional communication. Clinicians explained the tool would be useful to guide meaningful discussions between colleagues or when asked by a colleague about a particular condition.

“I am very grateful in the environment that I work in where I feel that would lead to a conversation. I hope that that is the purpose of this tool is that it leads to conversations about the clinical application of it…I think through conversations about why things are the way they are; there is a certain curiosity amongst my peers that there would be a discussion and we would have a discussion about how we would implement it. So I like it as a, let’s call it, a conversation starter… So that is how I would use it.” – Clinician 8 (male; 2 years in practice)

Similarly, clinicians discussed that the tool could be used to guide interprofessional communication about the care provided to patients. The tool could be shared among healthcare providers to discuss the care being provided to the patient.

“I think it is great…it is a good little pathway. Even to share with some other colleagues like a personal trainer to be able to, if I’m seeing one of their patients, to be able to say, ‘Hey, this is why I am doing this.’ So even to give it to, like I said, allied health professionals is a good idea.” – Clinician 6 (female; 12 years in practice)

Conversely, one clinician expressed the perception that the use of the care pathway as a communication tool may be unlikely.

“So to try and get it so that one clinician will talk to another clinician about it and say ‘hey this is great, I use this tool, you should start using it too’, I just don’t see chiropractors as using it very much.” – Clinician 1 (male; 23 years in practice)

### Theme: patient education

Clinicians reported that the care pathway might be a useful resource for their patients. They described using it as a visual tool to demonstrate where a patient is categorized within the care pathway, the available care options, and as a supportive resource to authenticate their treatment plan. Clinicians discussed that having such a visual tool may assist healthcare providers to engage patients and promote meaningful discussion about their care.

“One of the main kind of things that I saw myself using this for was not really for myself but more so for patient education … if this was a bit more of a piece of art, I would be happy to show oh here we go patients, take a look at this, this is what we are doing and this is why you can feel confident.” – Clinician 9 (male; 1 year in practice)

During the review of themes, one clinician contested the use of the care pathway for the purpose of an educational tool for patients. They discussed that the purpose of developing the care pathway is for clinicians, and considered use for patient education inappropriate.

“I don’t love the patient education theme. I understand that other participants said they would use it to show patients where they fit in the algorithm but I don’t think this guideline is meant for that purpose. I think this is a tool that should strictly be used for clinician use. Sure clinicians can pull the information and inform patients. I think that is very important and a key facet of knowledge transfer but I do not think one of the main purposes of this pathway is that it should be used as an infographic to educate patients.” -Clinician 8 (male; 2 years in practice)

### Research objective 2: end-user perceptions about the practicality of implementing the online care pathway into practice settings

Clinicians were asked to describe their thoughts regarding the practicality of trying to implement the care pathway into practice settings. Specifically, clinicians described enablers and barriers that may influence its implementation. Four themes arose through discussion.

### Theme: agreement with the care pathway

Clinicians alluded that they would be more apt to adopt the information into their practice if they agreed with the information presented in the care pathway. Similarly, clinicians would consider adopting the clinical pathway if the information presented closely resembled how they currently practiced.

They [clinicians] are going to focus on what they know and what they are comfortable with and what they have been taught. So regardless of whether or not they actually follow the recommendations, tough to say, I feel like some people would be kind of maybe stuck in their ways.” –Clinician 8 (male; 2 years in practice)

Conversely, if the clinician disagrees with the presented recommendations or if it differs from their current practice behaviours, they may be less motivated to adopt the information. One clinician described the dilemma of disagreeing with recommendations presented in the care pathway but held value in conceptually representing themselves as an evidence-based clinician.

“Now whether they agree or disagree with the data and the evidence; I mean, I was surprised by certain recommendations made and I made a comment and said, okay well I agree with this, I don’t agree with this but at the end of the day I know even if I don’t agree with it that is evidence right. So, I am at a crossroads. Am I going to be an evidence-based practitioner and follow the data or am I going to go off and be a cowboy.” – Clinician 8 (male; 2 years in practice)

### Theme: influence of clinician training

Clinicians expressed that the basic understanding of how to implement new evidence into practice should be a skill taught during the training to become a clinician and that trying to teach clinicians to do this after years in practice may not be successful.

“I think with these guidelines and these pathways you’re trying to facilitate a thought process so that people can optimize their skill set and their knowledge base. But you have to go backwards in their undergrad to do that because if you don’t, you’re spending a lot more time trying to teach them how to implement the guideline and it almost comes to a point where it’s a skill unto itself and then you have to execute on the guideline. That is just too much work, it is arduous.” – Clinician 4 (male; 13 years in practice)

When there is a discrepancy between what was learned and what is presented as new information (i.e., the care pathway); the acceptance of the new information/evidence may be limited.

“The reality is, people are going to insert their own personal opinions and styles but if you show them a more simplistic path or simplistic set of choices they will say ‘oh I do that already’ or ‘oh I should add that’”- Clinician 2 (male; 16 years in practice)

Further, a clinician’s experience and comfort level with sourcing and appraising research may influence the use of the care pathway in practice. While some clinicians may have experience with appraising research and how to integrate it into practice, other clinicians may lack this knowledge.

“In my particular clinical situation, I won’t have an older chiropractor who I go and ask for help from. This [the care pathway] is where I am going to get my information from other than messaging some of my colleagues and gathering information that way.” – clinician 9 (male; 1 year in practice)

### Theme: role of opinion leaders

Many professions, such as chiropractic, have opinion leaders who are regarded as trustworthy sources of information. These individuals are often seen as influential persons who can facilitate the uptake of evidence into clinical practice. Participants discussed the concept of having opinion leaders and their influential role in facilitating the implementation of the care pathway.

“…what we do know is that when people feel that some of their colleagues are part of this, people they know and respect and are champions and leaders, they are more likely to think that this is for them.” –Clinician 5 (male; 14 years in practice)

### Theme: difficulties with replicability of recommended interventions

Some clinicians discussed the challenges with implementing new evidence into their practices. They reported that published research studies often lack clear reporting or description of specific interventions, making it difficult for clinicians to replicate the intervention in their practices.

“…in a rehab context for any condition, the literature is a disaster. There is not enough description of interventions in most studies to come to any reasonable answer to average out what intervention might be best for anything so we are kind of left up in the air that way.” – Clinician 2 (male; 16 years in practice)

One clinician addressed the issue of replicability because of limitations in available resources/equipment. For example, if the care pathway recommends a particular intervention requiring specialized equipment, only clinicians with access to this equipment will be capable of implementing the intervention. This is an important consideration with regard to developing care pathways as not all recommendations will be suitable for implementation in all care settings.

…if they don’t have a piece of equipment. So, if one of the things is ultrasound, if they don’t have it they can’t use it so that might be a barrier, right? – Clinician 6 (female; 12 years in practice)

### Research objective 3: end-user feedback and recommendations for improving the care pathway

Clinicians were asked to provide their feedback about the care pathway while participating in the Think Aloud activity. Comments and suggestions were summarized. Generally, clinicians appeared to find the layout of the care pathway manageable. Other design items such as colour, font size, and easy-to-find hyperlinks to additional information were also noted as positive aspects of the pathway. Several clinicians also indicated that the care pathway had sufficient information for clinical decision-making.

“I personally like the pathway and the way they are laid out because they are simple to use. It is not an algorithm that is all over the place…to make it nice and tight and compact and in a nice package to hand to people, it makes it a lot easier to digest.” – Clinician 6 (female; 12 years in practice)

Regarding design features, some clinicians did not like the colour scheme; they felt the colours were too muted and suggested additional colours and design elements to make it more appealing to users. They also suggested there was too much information on each page, some hyperlinks appeared to be broken (non-functional), and that the instructions for clicking on the decisions or statements of the care pathway to obtain more information were not intuitive and required further clarity.

Clinicians made several recommendations for improving clinical components of the care pathway. Clinicians suggested including additional information in the clinical evaluation, such as listing specific orthopaedic tests and their predictive likelihood ratios, and providing hyperlinks to other care pathways and resources for patients who may present with other pathologies or conditions.

“The other key to this is it can’t not just be for shoulder, this has to be something structured that has continuity with other joints.” – Clinician 4 (male; 13 years in practice)

Clinicians suggested that prioritizing the list of recommendations/interventions from the guideline may be helpful. They provided several suggestions to improve clarity to the recommended interventions, such as including additional details of the recommended exercises with links to illustrative pictures and related articles. One clinician recommended using patient lifestyle education, such as smoking cessation. Finally, the care pathway indicates that clinicians should refer to a physician if the patient had an incomplete recovery following the management of chronic symptoms (Fig. 1). Several clinicians reported that this wording may be inappropriate given presumed limitations to access or expertise. Instead, a referral to an ‘appropriate healthcare provider,’ such as an orthopaedic surgeon, nurse practitioner, physical therapist, or sports chiropractor, may be preferable.

## Discussion

Our study explored end-user (clinician) perceptions of the usefulness and practicality of implementing a care pathway to manage shoulder pain. Additionally, we solicited feedback and recommendations for improving the care pathway. Our study included nine clinicians. We found that clinicians believed the care pathway could be useful at various levels, including education (students and interns), for early career clinicians, and as a reminder of information for certain, less familiar conditions. Clinicians also considered the care pathway to be useful for engaging patients and for facilitating interprofessional communication. Conversely, some clinicians did not perceive that the care pathway would be a useful tool to experienced clinicians because, from their perspective, the care pathway did not highlight any new or changing evidence. Our findings suggest the care pathway may be best suited as an educational resource for early career clinicians or as a part of curriculum in educational settings. The relationship between a clinicians’ level of experience and the perceived use of the care pathway is unclear. However, it is possible that clinicians who have been in practice longer may perceive clinical experience as outweighing of scientific evidence [36, 37]. Future research should explore how the pathway could be modified to also benefit experienced clinicians.

With regard to the practicality of implementing the care pathway into clinical practice settings, clinicians described factors associated with implementation; described as barriers and facilitators. Our analysis of clinicians’ perceptions of usefulness demonstrates that the care pathway may address some personal barriers (i.e., clinicians thought the care pathway was a useful tool and saw themselves using it in a variety of ways). On the other hand, clinicians’ perceptions about the practicality of implementing the care pathway highlights systemic and contextual barriers as also demonstrated in previous studies (i.e., the need for opinion leaders, lack of time, lack of resources) [5, 6]. Addressing such barriers may have been due to our iKT approach; by including input from the clinicians throughout the development of the clinical tool we may have been able to directly address potential personal barriers to implementation. Though many of the clinicians who participated play other roles outside of their clinical responsibilities (e.g., education, research, administrative), it is possible that the inclusion of other key stakeholders (i.e., patients,) would have provided additional recommendations to address other contextual and systemic barriers implementing the clinical tool.

Our findings support the results of previous qualitative studies that identified barriers and facilitators for using and implementing clinical pathways in healthcare settings [4, 38–40]. For example, a qualitative study by Reyneke, et al. [4] aimed to identify barriers and facilitators of general practitioners using clinical pathways in a primarily urban health region in New Zealand. The care pathways were web-based flow diagrams directing practitioners to treatment modalities available in the community. In general, practitioners agreed that there is merit in using the care pathways to improve patient care and outcomes; however, they also identified significant barriers to its implementation. For example, practitioners described feelings of information overload, challenges with learning and using new technology, as well as losing their autonomy over clinical decision-making, among others [4]. Additional studies of varying healthcare disciplines (surgeons, nurses, and anesthesiologists [38], physicians, hospital administration, and nurses [39], nurses, ICU physicians, transplant coordinator, social workers, senior physicians, senior surgeons [40]) report similar findings as well. Our study comprised primarily of chiropractors, however, our findings related to the barriers of care pathway acceptability and practicality of implementation are similar to those involving other healthcare professions.

Strategies to promote implementation need to be multifaceted and context-specific, considering individual, systemic, and contextual factors [3, 5–7, 41]. While clinicians appear to play a significant role in implementing evidence-based care, other stakeholders also play significant roles. Future research should consider including all individuals who play a role in implementing evidence into practice. These stakeholders may include, but are not limited to, clinic support staff, educators, opinion leaders, governing associations, electronic charting software developers, and policymakers. A study by De Allegri, et al. [38] explored the experiences of interdisciplinary staff members (surgeons, anaesthesiologists, nurses) at all hierarchy and seniority levels of a surgery department in a German hospital aiming to develop and implement care pathways for the management of kidney transplantation, thoracic surgery and colorectal surgery. Interestingly, this study highlights an important phenomenon whereby the hierarchical structure of the hospital setting may have marginalized the participation of some individuals, in this case nurses. Though this study does not provide suggestion to overcome this barrier, it is of particular importance when considering the implementation of care pathways into interdisciplinary settings. Future research should consider aiming to identify strategies to overcome barriers of hierarchical structure in healthcare settings as we know that the implementation of care pathways in healthcare settings is best facilitated by all those involved in its application. Finally, patients may also play an important role in the implementation of evidence-based practice guidelines. With information readily at their fingertips, patients may be better informed and state their preferences for specific treatments that may or may not be recommended [42–44]. Some clinicians considered that the care pathway could also be used as an educational tool for patients. Though this sentiment was not agreed upon by all participating clinicians, future research should engage patients as stakeholders to explore their perspective on the perceived usefulness and potential implementation strategies of the tool as a patient-specific educational resource.

Our study is situated within a larger project, whereby end-users co-developed an evidence-based care pathway to facilitate the clinical management of shoulder pain in adults. In a previous publication, we describe clinicians’ experiences of co-producing the care pathway and their perceptions of participating in knowledge tool development [21]. Collectively, our findings provide a guide for engaging clinicians in integrated knowledge translation (iKT) endeavors and co-producing clinical resources [45–51].

### Strengths and limitations

Our study has several strengths. We followed a sound qualitative methodology to guide the conduct of our study. Using this framework, themes were sequenced and linked to create a web of thematic connections insightfully describing the overall phenomena for each of our research objectives. To improve trustworthiness of our data and externally audit our analysis, we implemented member checking at two time points: 1) following each individual interview and 2) review of the overall analysis (mind maps, themes, and definitions). We also acknowledge some limitations. The transferability of our findings may be impacted by our inability to recruit a balanced sample across professions. It is unknown whether our findings may have differed with a more professionally-diverse (i.e., profession, years in practice, healthcare setting) group of clinicians. However, within our study population, it appears that we were able to reach saturation. Additionally, our sampling method (recruitment from professional networks) may have influenced our findings by yielding more homogeneous perspectives. Finally, the research team’s assumptions and biases were not formally assessed which may have, inadvertently, perpetuated bias within our findings. For example, we did not specifically discuss how the research teams’ personal beliefs and biases may have influenced the process of data collection and analysis (reflexivity). However, in the analysis stage, we aimed to limit these biases through bracketing and by having the same experienced interviewer lead all interviews using an interview guide, and a second researcher was present for all interviews. Furthermore, three researchers independently coded themes and we used member checking to confirm overall thematic analyses.

## Conclusion

Exploring the perceptions of end-users about the perceived use and practicality of implementing care pathways in healthcare settings provides tangible instruction to improve the care pathways themselves as well as their implementation strategies. While research continues to explore the individual, systemic and contextual barriers present in our healthcare settings, our methods of end-user involvement and collaboration may help to support and strengthen future strategies for implementation.

## Supplementary Information

**Additional file 1.** Interview Guide

## Data Availability

The datasets generated and/or analyzed during the current study are not publicly available due to ethical standards protecting study participation; however, can be made available from the corresponding author on reasonable request.

## Abbreviations

iKT: Integrated knowledge translation
COREQ: Consolidated Criteria for Reporting Qualitative Research Checklist
CCGI: Canadian Chiropractic Guideline Initiative
